# Changes in Diet Quality over 10 Years and Subsequent Mortality from Cardiovascular Disease in the Multiethnic Cohort Study

**DOI:** 10.3390/nu15153482

**Published:** 2023-08-07

**Authors:** Minji Kang, Carol J. Boushey, Yurii B. Shvetsov, Veronica W. Setiawan, Hee-Young Paik, Lynne R. Wilkens, Loïc Le Marchand, Song-Yi Park

**Affiliations:** 1 Department of Food and Nutrition, Duksung Women’s University, Seoul 01369, Republic of Korea; 2 Cancer Epidemiology Program, University of Hawaii Cancer Center, Honolulu, HI 96813, USA; cjboushey@cc.hawaii.edu (C.J.B.); yshvetso@cc.hawaii.edu (Y.B.S.); lynne@cc.hawaii.edu (L.R.W.); loic@cc.hawaii.edu (L.L.M.); spark@cc.hawaii.edu (S.-Y.P.); 3 Department of Population and Public Health Sciences, Keck School of Medicine and Norris Comprehensive Cancer Center, University of Southern California, Los Angeles, CA 90089, USA; vsetiawa@med.usc.edu; 4 Department of Food and Nutrition, Seoul National University, Seoul 08826, Republic of Korea; hypaik@snu.ac.kr; 5 Center for Gendered Innovations for Science and Technology Research (GISTeR), Korea Federation of Women’s Science & Technology Associations, Seoul 06130, Republic of Korea

**Keywords:** cardiovascular disease mortality, change in diet quality, multiethnic population, cohort study

## Abstract

This study investigated how diet quality changes over a ten-year period, assessed using the following four diet quality indexes, the Healthy Eating Index-2015 (HEI-2015), Alternative Healthy Eating Index-2010 (AHEI-2010), alternate Mediterranean Diet (aMED), and Dietary Approaches to Stop Hypertension (DASH), were related to mortality from cardiovascular disease (CVD) in the Multiethnic Cohort Study. The analysis included 61,361 participants who completed both the 1993–1996 baseline survey and the 2003–2008 10-year follow-up surveys. Over the mean follow-up period of 13 years after the 10-year survey, 4174 deaths from CVD were identified. Hazard ratios (HRs) and 95% confidence intervals (CIs) were estimated using multivariable Cox models. Increases in diet quality scores were associated with a reduced risk of CVD mortality for all indexes: HRs per one SD increment of 0.94 to 0.99 (HR (95% CI), 0.96 (0.92–1.01) for HEI-2015, 0.96 (0.91–1.01) for AHEI-2010, 0.99 (0.94–1.04) for aMED, and 0.94 (0.89–0.99) for DASH) in men and 0.88 to 0.92 (0.88 (0.84–0.92) for HEI-2015, 0.90 (0.85–0.95) for AHEI-2010, 0.89 (0.84–0.95) for aMED, and 0.92 (0.87–0.96) for DASH) in women. The inverse association generally did not vary by race and ethnicity, age, body mass index, smoking, and hypertension in each sex. Our findings suggest that improving diet quality and maintaining a high-quality diet over time may help reduce the risk of CVD mortality and could also be beneficial for those at higher risk of CVD.

## 1. Introduction

Cardiovascular disease (CVD) is the primary cause of death in the United States [1], and maintaining a healthy diet is a crucial factor that can be modified to reduce the risk of CVD [2]. Several indexes have been developed to evaluate overall diet quality, which include the Healthy Eating Index (HEI), Alternative Healthy Eating Index (AHEI), alternate Mediterranean Diet (aMED), and Dietary Approaches to Stop Hypertension (DASH) scores [3,4,5,6]. A growing body of evidence indicates that diets of high quality, as evaluated using a *priori* indexes are linked with a lower risk of CVD [7,8,9,10,11,12]. A meta-analysis of 34 cohort studies revealed that adopting a high-quality diet, as defined by HEI, AHEI, and DASH scores, was associated with a 22% lower risk of CVD mortality or incidence [12]. Specifically, for individuals with the highest diet quality compared to those with the lowest diet quality, the relative risk (RR) was 0.78, with a 95% confidence interval (CI) of 0.76–0.80 [12]. In addition, in a meta-analysis of 29 observational studies, following the Mediterranean diet was related to a 19% decreased risk of CVD mortality or incidence (RR 0.81, 95% CI 0.74–0.88) [11].

Although dietary habits may change over time, there have only been a few studies that have explored diet quality change in relation to CVD risk or mortality [13,14]. A prospective study reported that a 20-percentile increase in AHEI, aMED, and DASH scores over 12 years was significantly associated with a 7–15% reduction in the risk of CVD mortality among men and women primarily of White ancestry [14]. This study investigated the associations between changes in diet quality over ten years, as assessed using four indexes (HEI-2015, AHEI-2010, aMED, and DASH) and subsequent CVD mortality in multiethnic populations. Our investigation involved analyzing these associations in both men and women and considering potential variations based on race and ethnicity, age, body mass index (BMI), smoking, and history of hypertension.

## 2. Materials and Methods

### 2.1. Study Population and Design

The MEC is a large prospective cohort study established to investigate lifestyle factors, including diet, and their impact on health-related outcomes. In 1993–1996, a cohort consisting of 215,251 individuals, aged 45 to 75, living in Hawaii or California was formed [15]. The cohort was composed mainly of individuals from five races and ethnicities: African American, Japanese American, Latino, Native Hawaiian, and White [15]. Upon entering the cohort, participants were asked to complete a comprehensive 26-page self-administered mail questionnaire. This questionnaire encompassed a quantitative food frequency questionnaire (QFFQ) to capture detailed information about participants’ dietary habits. A total of 98,214 participants responded to a 10-year follow-up survey repeating the QFFQ in 2003–2008. The study was approved by the institutional review boards at the University of Hawaii (CHS9575) and the University of Southern California (HS-17-00714).

In our current study, we excluded participants who responded that they did not belong to one of the five racial and ethnic groups (*n* = 5246) and those who responded with a history of angina, stroke, or heart attack at either the baseline survey or the 10-year follow-up survey (*n* = 14,466). Furthermore, participants with implausible dietary intake at the baseline or the 10-year follow-up were excluded from the analysis (*n* = 5930). We calculated a robust standard deviation (RSD) of energy intake using the middle 80% of the normal distribution. Subsequently, we excluded participants whose total energy intake less than the mean—3RSD or greater than the mean + 3RSD. A similar process was implemented for intakes of carbohydrate, protein, and fat. We further removed participants who had missing or invalid (<15 kg/m^2^ or ≥50 kg/m^2^) BMI at either survey (*n* = 3137) or who did not provide smoking information at the 10-year follow-up (*n* = 8229). Lastly, participants with missing values in covariates, including education, marital status, physical activity, and menopausal hormone therapy use (for women only), were excluded (*n* = 2965), which resulted in a total of 61,361 participants (26,624 men and 34,737 women) in the final analysis.

### 2.2. Dietary Assessment and Calculation of Dietary Quality

Dietary intakes were obtained using the QFFQ encompassing more than 180 food items at the baseline and 10-year follow-up surveys [15]. Data from 3-day measured food records were used to develop the baseline QFFQ, which was then validated in a calibration study. The calibration study demonstrated satisfactory correlations (ranging from 0.57 to 0.74) for nutrients as densities between the QFFQ and three 24 h dietary recalls (24HDR) [16]. The 10-year follow-up QFFQ remained mainly comparable to the baseline QFFQ, with a few primarily minor changes. These modifications entailed changes in the design, incorporating new food products into the food lists and providing examples for each item. In a reproducibility study, we observed high correlations (ranging from 0.70 to 0.74) for nutrient densities between the baseline and 10-year QFFQs [17]. Daily energy and nutrient intakes were computed utilizing MEC food composition tables [18,19].

As part of the Dietary Patterns Methods Project [8,20], four predefined diet quality indexes (including HEI-2015, AHEI-2010, aMED, and DASH scores) were calculated for the MEC participants. These indexes were then used to assess changes in diet quality over ten years. Briefly, the HEI-2015 is a scoring system with a theoretical score range of 0 to 100 points, encompassing 13 components. This is a thorough evaluation of how well one follows the Dietary Guidelines for Americans 2015–2020 [21,22]. These 13 components of 9 adequacy components assess the intakes of nutrients and food groups based on “total fruits”, “whole fruits”, “total vegetables”, “greens and beans”, “whole grains”, “dairy”, “total protein foods”, “seafood and plant proteins”, and “fatty acids”. Additionally, four moderation components evaluate the consumption of certain nutrients and food items to be limited, including “refined grains”, “sodium”, “added sugars”, and “saturated fats”. The AHEI-2010 is a scoring system ranging from 0 to 110 points, comprising 11 components. It was developed based on extensive clinical and epidemiologic research, identifying foods and nutrients that have shown to be predictive of chronic disease risk [4,23]. The 11 components of the AHEI-2010 are as follows: “vegetables”, “fruit”, “whole grains”, “sugar-sweetened beverages and fruit juice”, “nuts and legumes”, “red/processed meat”, “trans fat”, “long-chain (*n*-3) fats (EPA + DHA)”, “PUFA”, “sodium”, and “alcohol”. Each component is scored from 0 (worst) to 10 (best). The aMED is a scoring system ranging from 0 to 9 points, adapted from the Mediterranean diet score, associated with a decreased risk of chronic disease [5,24]. The aMED consists of nine components, each scored from 0 (worst) to 1 (best). The nine components of the aMED are as follows: “vegetables”, “legumes”, “fruit”, “nuts”, “whole grains”, “red and processed meats”, “fish”, “ratio of monounsaturated to saturated fat”, and “ethanol”. The DASH (8 to 40 points with eight components) score is based on food and nutrients to aid in the management of hypertension [6,25]. It consists of eight components, each scored on a scale from 1 (worst) to 5 (best). The eight components of the DASH diet are as follows: “fruits”, “vegetables”, “nuts and legumes”, “whole grains”, “low-fat dairy”, “sodium”, “red and processed meats”, and “sweetened beverages”. For all four indexes, higher scores reflect a higher-quality diet. To ensure that differences in the scores between baseline and 10-year QFFQs reflected individual changes in diet, baseline cut points were applied to the 10-year follow-up indexes. For indices that necessitated population-specific quantiles for scoring, such as the median for aMED, the quintile for DASH, or the decile for AHEI-2010, the corresponding baseline cut points were used [26].

### 2.3. Death Ascertainment

Through linkage with the state death certificate records in Hawaii and California, as well as the National Death Index up to 31 December 2019, deaths were identified. Deaths attributed to CVD were classified using the International Classification of Diseases, Ninth Revision (ICD-9) codes 390–448 or Tenth Revision (ICD-10) codes I00–I78 and G45 [27,28]. Over the mean follow-up period of 13.0 years after the 10-year follow-up survey, we identified 4174 deaths from CVD among the 61,361 eligible participants.

### 2.4. Statistical Analysis

Diet quality changes over ten years were analyzed using two approaches. The first approach involved assessing changes in diet quality by calculating the difference in scores between the 10-year follow-up and the cohort entry. These changes were then examined based on the standard deviation (SD) of change for each index. This approach was necessary since the scales of the diet quality indexes used in the study differed, and using the standard deviation allowed for a more standardized comparison. The SD of change in the score for men and women was 9.8 and 9.9 for HEI-2015 and 10.0 and 9.5 for AHEI-2010, respectively, while it was 1.9 for aMED and 4.0 for DASH in both men and women. Diet quality changes were modeled in continuous increments of 1 SD or categorically: (1) greatest decrease (of ≥1 SD), (2) moderate decrease (of 0.5–1 SD), (3) stable (absolute change of <0.5 SD, reference), (4) moderate increase (of 0.5–1 SD), and (5) greatest increase (of ≥1 SD). In the second approach, four trajectory categories for score changes were defined based on the median values of each index. The categories were defined as follows: (1) consistently low (participants whose scores remained below the median on both surveys), (2) high to low (participants who experienced a change from having dietary scores above the median in the baseline survey to below the median in the 10-year follow-up), (3) low to high (participants who changed from having dietary scores below the median in the baseline survey to above the median in the 10-year follow-up), and (4) consistently high (participants whose dietary scores remained above the median at both surveys). The consistently low group served as the reference.

Hazard ratios (HRs) and 95% confidence intervals (CIs) for CVD mortality were estimated using Cox proportional hazard models, with age serving as the time metric. These estimates were based on changes in diet quality, and analyses were conducted separately for men and women. The following variables were adjusted: race and ethnicity (African American, Japanese American, Latino, Native Hawaiian, and White), age group (<65, 65–75, and ≥75 years), BMI group (<25, 25–30, and ≥30 kg/m^2^) [29], and history of hypertension (yes, no) as strata variables, and marital status (married, not married), education (≤high school, vocational school or some college, and ≥graduated college), physical activity (<0.5, 0.5–1.3, and >1.3 h spent in moderate or vigorous physical activity per day), menopausal hormone therapy use (never, ever) for women only, BMI change between two surveys (kg/m^2^), total energy intake (kcal/day), and baseline diet quality score as covariates, and smoking model [30]. In the analysis of the HEI-2015 and DASH diet, the models were additionally adjusted for alcohol consumption (g/day). This adjustment was necessary because alcohol intake is not included as a component of these two indexes. All of the covariates used in the analysis were obtained from the 10-year follow-up survey, except for race and ethnicity, and education, which were obtained from the baseline questionnaire. For the trajectory of the change in diet quality, baseline dietary scores were not adjusted because they were incorporated into the trajectory definition. The proportional hazards assumption was tested using the Schoenfeld residual method, and the results indicated that the assumption was met [31]. Subgroup analyses were performed to examine whether associations varied by race and ethnicity, age, BMI, smoking status, and history of hypertension within the sexes. Tests for heterogeneity by sex and subgroups were based on the Wald statistics for cross-product terms of continuous variables for diet quality change and subgroup indicators. All statistical tests were two-sided. The SAS statistical software version 9.4 was used to conduct all statistical analyses (SAS Institute, Cary, NC, USA).

## 3. Results

The mean age was 68.4 (SD 8.3) years in men and 68.3 (SD 8.3) years in women at the 10-year follow-up survey (Table 1). Over ten years, the HEI-2015 score improved in men (65.8 to 68.9) and women (69.6 to 72.6). Men and women with the greatest increase in HEI-2015 scores tended to be younger and of Japanese American ethnicity. Additionally, they were less likely to be currently smoking at the 10-year follow-up and less likely to have a hypertension history than those with stable HEI-2015. On the other hand, men and women with the greatest decrease in HEI-2015 tended to be older and of African American or White ethnicity. They were more inclined to be current smokers at the 10-year follow-up and had a higher prevalence of prior hypertension. See Supplementary Materials Tables S1–S3 for characteristics related to the change in the AHEI-2010, aMED, and DASH scores.

Table 2 shows the HRs and 95% CIs for CVD mortality based on the 10-year change in the HEI-2015, AHEI-2010, aMED, and DASH scores. Individuals who experienced the greatest decrease (>1 SD) in their diet quality scores had a higher risk of CVD mortality compared to those who maintained stable scores; in men, the HR (95% CI) was 1.13 (0.97–1.31) for HEI-2015, 1.12 (0.96–1.30) for AHEI-2010, 1.12 (0.97–1.29) for aMED, and 1.38 (1.18–1.62) for DASH, and in women, 1.42 (1.24–1.62) for HEI-2015, 1.33 (1.16–1.52) for AHEI-2010, 1.29 (1.12–1.48) for aMED, and 1.30 (1.11–1.51) for DASH. An increase in the quality of one’s diet, measured in one SD, was found to be associated with a lower risk of mortality from CVD. The HR (95% CI) was 0.96 (0.92–1.01) for HEI-2015, 0.96 (0.91–1.01) for AHEI-2010, 0.99 (0.94–1.04) for aMED, and 0.94 (0.89–0.99) for DASH in men. In women, a significant risk reduction was observed across the four indexes with an HR range of 0.88 to 0.92 (0.88 (0.84–0.92) for HEI-2015, 0.90 (0.85–0.95) for AHEI-2010, 0.89 (0.84–0.95) for aMED, and 0.92 (0.87–0.96) for DASH). No heterogeneity by sex was detected, except for HEI-2015, with a stronger association in women than men (*P* for heterogeneity = 0.0066).

In subgroup analyses by race and ethnicity, age, BMI, smoking, and history of hypertension (Table 3), the inverse association per one SD increment generally did not vary across the subgroups in each sex, but it appeared to be more consistent in women than in men overall. For example, the risk reduction per one SD increment in the AHEI-2010 was significant in men with a history of hypertension but not in men without a history of hypertension (*P* for heterogeneity = 0.0059). In women, the HRs for one SD increment were below 1 in most subgroups by race and ethnicity, age, BMI, smoking, and history of hypertension, except for Latinos. However, in both men and women, there was no indication of heterogeneity in the relationship across subgroups (all *P*’s for heterogeneity > 0.00125 with Bonferroni correction).

**Figure 1:**
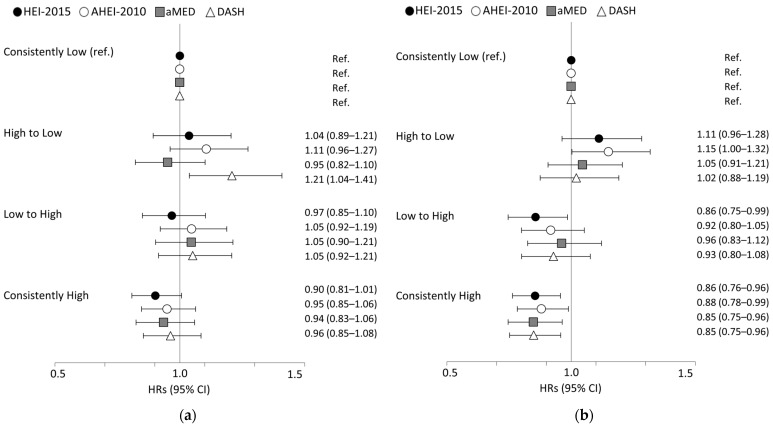
HRs (95% CI) for cardiovascular disease mortality according to change in diet quality over 10 years in the Multiethnic Cohort Study: (**a**) men; (**b**) women. AHEI, Alternative Healthy Eating Index; aMED, alternate Mediterranean Diet score; DASH, Dietary Approaches to Stop Hypertension; HEI, Healthy Eating Index. Changes in diet quality were categorized into four groups based on the median scores of each index at baseline as follows: (1) consistently low (participants whose scores remained below the median on both surveys); (2) high to low (participants who experienced a change from having dietary scores above the median in the baseline survey to below the median in the 10-year follow-up); (3) low to high (participants who changed from having dietary scores below the median in the baseline survey to above the median in the 10-year follow-up); (4) consistently high (participants whose dietary scores remained above the median at both surveys). Adjusted for race and ethnicity, age, BMI, history of hypertension, education, marital status, physical activity, menopausal hormone therapy uses for women only, BMI change between two surveys, total energy intake, and smoking model. For HEI-2015 and DASH, the models were further adjusted for alcohol intake. All variables were from the 10-year follow-up survey, except for race and ethnicity and education from the baseline questionnaire.

Figure 1 shows the associations between diet quality trajectory over ten years and the risk of CVD mortality. When comparing men who continued to have low diet quality, men with a decline from high to low quality were found to have an increased risk of CVD mortality for DASH (1.21 (1.04–1.41)). Women with consistently high-quality diets had a lower risk of CVD mortality across indexes (HRs of 0.85 to 0.88), and those with improvement in diet quality also showed a reduced risk, especially for HEI-2015 (0.86 (0.75–0.99)), while a decline from high to low quality was related to an increased risk for AHEI-2010 (1.15 (1.00–1.32)).

## 4. Discussion

The current study investigated the associations between diet quality changes over ten years, as measured using the HEI-2015, AHEI-2010, aMED, and DASH, and the subsequent risk of CVD mortality in a large multiethnic population. Maintaining a high-quality diet and improving its quality were related to lower the risk of CVD mortality. In contrast, a reduction in diet quality was linked to a higher risk of CVD mortality in both men and women. The inverse association for improvement in diet quality generally remained consistent across different subgroups, including race and ethnicity, age, BMI, smoking, and history of hypertension.

Prospective studies demonstrated that a higher quality diet, measured at baseline with a priori indexes, was associated with a reduced CVD risk [7,10,11,12,23]. Previous studies in the MEC also showed inverse associations between baseline diet quality and the risk of mortality from CVD [8,9] and stroke specifically [32] for all dietary indexes. However, longitudinal diet quality changes have rarely been studied in relation to CVD [13,14]. In the Nurses’ Health Study (NHS) and the Health Professionals Follow-Up Study (HPFS), the greatest improvement in the AHEI and aMED over four years was associated with a 7–9% lower CVD risk over the next 20 years, compared with stable diet quality [13]. Another study from the NHS and the HPFS found that a 20-percentile increase in the AHEI, aMED, and DASH over 12 years was related to a 7–15% reduction in the risk of subsequent death from CVD [14].

The associations found in the MEC were largely consistent across the dietary indexes, showing that increasing or retaining high scores over time was consistent with reducing the risk of mortality from CVD. These results could potentially indicate an enhancement in the diet quality of the participants in the MEC study, focusing on components commonly emphasized in the diet quality indexes. Over ten years, the average scores for various food groups, including vegetables, fruits, dairy, and protein foods, remained stable or slightly increased for both men and women [26]. In addition, although the indexes used in the current study differ in composition and scoring, all emphasize a diet containing a variety of vegetables, whole grains, fruits, fish, and lean meats [2,3,4,5,6,21,22,23,24,25], as well as foods rich in nutrients such as mono- and polyunsaturated fatty acids, antioxidant vitamins, minerals, and dietary fiber, which were identified as having a beneficial effect on CVD prevention [20,33,34]. On the other hand, the indexes emphasize consuming less saturated fat, trans fat, added sugars, and sodium, as these have been associated with an elevated risk of CVD death [33,35,36].

A few prospective studies have found that diet quality is associated with reduced CVD outcomes among individuals at high risk of CVD [37,38,39]. For example, a study involving approximately 31,500 individuals diagnosed with hypertension revealed that those with higher diet quality, assessed using a modified AHEI, had a 17% reduced risk (with an HR 0.83, 95% CI 0.74–0.92 for the highest vs. lowest quintiles) of experiencing a primary composite outcome. This composite outcome consisted of CVD death, myocardial infarction, stroke, or congestive heart failure [37]. Consistent with the previous studies of the population at high risk for CVD, our results showed the beneficial effect of improving diet quality for individuals with prior hypertension who had a twice more increased risk of CVD death than those without hypertension. These results strengthen the incentive for individuals at higher risk of CVD to adopt and maintain healthy dietary patterns.

This study has several strengths, including its prospective design, the considerable number of participants with diverse racial and ethnic backgrounds, repeated dietary assessment over ten years, and comprehensive data on various potential confounding factors. In addition, the QFFQ was specifically tailored to include ethnic-specific foods [15], which allowed for relevant comparisons according to race and ethnicity. Nevertheless, there are some limitations that need to be considered. Dietary data obtained through a self-administered QFFQ are prone to nondifferential measurement errors. These errors are frequently observed in cohort studies and can lead to a weakening of the observed risk associations [40]. While the current analysis excluded participants with prevalent heart disease, we could not entirely rule out the possibility that changes in diet quality after the 10-year follow-up period were influenced by underlying illnesses or the development of medical conditions during this time. However, the exclusion of CVD deaths within the first two years since the 10-year follow-up to minimize the reverse causality bias led to similar results. Lastly, the findings are derived from those who responded to the 10-year follow-up survey, representing 45% of the cohort members. These respondents exhibited certain characteristics, such as being younger, having a higher representation of Japanese American or White ethnicity, being nonsmokers, being more educated, and being less obese compared to nonrespondents. Thus, there is a possibility of selection bias, which may limit the generalizability of the study’s results to the entire cohort population.

## 5. Conclusions

In summary, our findings suggest that improving diet quality and maintaining a high-quality diet over time may help reduce the risk of CVD mortality and could also be beneficial for those at higher risk of CVD.

## Figures and Tables

**Table 1:** Characteristics of participants according to a change in the Healthy Eating Index (HEI)-2015 over 10 years in the Multiethnic Cohort Study.

	All	Change in Healthy Eating Index (HEI)-2015 ^1^				
		Greatest Decline	Moderate Decline	Stable	Moderate Increase	Greatest Increase
Men, *n*	26,624	2262	2886	10,355	4815	6306
HEI-2015 score at baseline	65.8 ± 10.3	73.9 ± 8.9	70.5 ± 9.2	67.2 ± 9.7	64.1 ± 9.5	59.7 ± 9.1
HEI-2015 score at 10-year follow-up	68.9 ± 10.6	59.2 ± 9.2	63.5 ± 9.2	67.5 ± 9.8	71.3 ± 9.4	75.6 ± 8.9
Age at baseline (years)	57.4 ± 8.3	59.4 ± 8.6	58.2 ± 8.5	57.8 ± 8.4	57.0 ± 8.2	55.9 ± 7.7
Age at 10-year follow-up (years)	68.4 ± 8.3	70.4 ± 8.6	69.1 ± 8.5	68.8 ± 8.4	67.9 ± 8.2	66.9 ± 7.7
Race and ethnicity (%)						
African American	7.9	12.3	9.9	8.0	7.1	6.0
Japanese American	36.2	24.8	28.0	34.6	39.2	44.2
Latino	18.5	19.1	20.3	19.0	17.9	17.1
Native Hawaiian	7.1	6.5	7.6	6.7	7.2	7.9
White	30.3	37.2	34.1	31.8	28.6	24.8
Education (%)						
≤High school	28.3	31.7	27.9	28.2	26.9	28.4
Vocational school	30.7	30.3	30.8	30.4	30.4	31.5
≥Graduated college	41.0	38.0	41.3	41.4	42.8	40.1
Body mass index at baseline (kg/m^2^)	26.4 ± 3.8	26.5 ± 3.9	26.4 ± 3.8	26.3 ± 3.7	26.3 ± 3.8	26.5 ± 3.8
Body mass index at 10-year follow-up (kg/m^2^)	26.7 ± 4.2	26.7 ± 4.5	26.7 ± 4.3	26.7 ± 4.2	26.6 ± 4.1	26.6 ± 4.2
Smoking status at baseline (%)						
Never	36.9	34.2	37.4	37.1	39.0	35.6
Former	50.0	52.8	49.1	50.4	47.9	50.3
Current	13.1	13.1	13.5	12.5	13.1	14.0
Smoking status at 10-year follow-up (%)						
Never	37.0	34.3	37.5	37.2	39.2	35.8
Former	55.8	57.3	54.2	55.4	54.0	58.1
Current	7.2	8.4	8.3	7.4	6.8	6.2
Physical activity at baseline (h/d)	1.5 ± 1.5	1.4 ± 1.5	1.5 ± 1.5	1.5 ± 1.5	1.5 ± 1.5	1.4 ± 1.5
Physical activity at 10-year follow-up (h/d)	1.7 ± 1.6	1.4 ± 1.6	1.6 ± 1.6	1.7 ± 1.6	1.7 ± 1.6	1.7 ± 1.6
Alcohol intake at baseline (g/day)	14.9 ± 28.3	16.0 ± 32.0	16.1 ± 28.3	15.2 ± 27.7	14.1 ± 26.6	14.1 ± 29.1
Alcohol intake at 10-year follow-up (g/day)	11.6 ± 20.5	9.5 ± 20.4	11.2 ± 20.8	11.7 ± 20.2	12.0 ± 20.4	11.8 ± 20.9
History of hypertension at either survey	52.5	55.0	52.6	52.1	52.9	52.0
Women, *n*	34,737	3152	3776	13,588	6335	7886
HEI-2015 score at baseline	69.6 ± 10.3	76.5 ± 8.7	74.3 ± 9.2	71.4 ± 9.7	68.1 ± 9.4	63.0 ± 9.0
HEI-2015 score at 10-year follow-up	72.6 ± 10.6	61.5 ± 9.1	67.1 ± 9.2	71.6 ± 9.7	75.4 ± 9.4	78.9 ± 8.6
Age at baseline (years)	57.3 ± 8.3	59.2 ± 8.5	58.6 ± 8.5	57.7 ± 8.3	56.9 ± 8.1	55.4 ± 7.8
Age at 10-year follow-up (years)	68.3 ± 8.3	70.2 ± 8.6	69.6 ± 8.5	68.7 ± 8.3	67.8 ± 8.1	66.5 ± 7.8
Race and ethnicity (%)						
African American	11.6	15.2	14.0	12.1	9.9	9.3
Japanese American	34.7	24.7	28.2	33.3	38.5	41.0
Latino	15.6	17.1	15.9	14.3	14.8	17.6
Native Hawaiian	7.7	7.5	7.8	7.5	8.2	7.7
White	30.5	35.5	34.0	32.7	28.6	24.3
Education (%)						
≤High school	33.1	35.5	35.1	32.7	31.7	33.0
Vocational school	32.2	32.5	32.5	32.2	32.4	31.8
≥Graduated college	34.7	32.0	32.4	35.1	35.9	35.2
Body mass index at baseline (kg/m^2^)	25.5 ± 5	26.0 ± 5.0	25.8 ± 5.1	25.5 ± 5.0	25.3 ± 4.9	25.5 ± 5.1
Body mass index at 10-year follow-up (kg/m^2^)	26 ± 5.4	26.6 ± 5.7	26.3 ± 5.6	25.9 ± 5.4	25.8 ± 5.3	25.9 ± 5.3
Smoking status at baseline (%)						
Never	61.4	56.3	60.3	60.6	62.7	64.5
Former	27.8	31.6	29.3	28.3	27.3	24.9
Current	10.8	12.1	10.4	11.1	9.9	10.6
Smoking status at 10-year follow-up (%)						
Never	61.6	56.5	60.4	60.7	62.9	64.7
Former	32.8	36.0	33.5	33.2	32.2	30.8
Current	5.7	7.5	6.1	6.0	5.0	4.5
Physical activity at baseline (h/d)	1.2 ± 1.3	1.3 ± 1.3	1.3 ± 1.3	1.3 ± 1.3	1.2 ± 1.3	1.1 ± 1.2
Physical activity at 10-year follow-up (h/d)	1.4 ± 1.5	1.3 ± 1.4	1.4 ± 1.4	1.4 ± 1.5	1.5 ± 1.4	1.4 ± 1.5
Alcohol intake at baseline (g/day)	4.8 ± 14	5.5 ± 15.1	5.2 ± 14.1	5.3 ± 14.9	4.5 ± 14.1	3.5 ± 11.7
Alcohol intake at 10-year follow-up (g/day)	4.1 ± 10.5	4.0 ± 11.6	4.1 ± 10.3	4.5 ± 11.1	3.9 ± 9.8	3.3 ± 9.8
History of hypertension at either survey	51.6	55.6	52.8	51.8	50.8	49.8

^1^ Greatest decline: ≥1 SD decrease; moderate decline: 0.5–1 SD decrease; stable: <0.5 SD change; moderate increase: 0.5–1 SD increase; greatest increase: ≥1 SD increase.

**Table 2:** HRs (95% CI) for cardiovascular disease (CVD) mortality according to change in diet quality over 10 years in the Multiethnic Cohort Study.

Diet Quality Change ^1^	Men (*n* = 26,624)		Women (*n* = 34,737)		*P* for Heterogeneity ^3^
	CVD Death	HR (95% CI) ^2^	CVD Death	HR (95% CI) ^2^	
HEI-2015					
Greatest decline	240	1.13 (0.97–1.31)	303	1.42 (1.24–1.62)	
Moderate decline	250	1.01 (0.88–1.17)	275	1.15 (1.00–1.32)	
Stable	858	1.00 (ref.)	812	1.00 (ref.)	
Moderate increase	360	0.99 (0.87–1.12)	320	0.95 (0.83–1.08)	
Greatest increase	425	1.01 (0.89–1.14)	331	0.89 (0.78–1.02)	
Per 1 SD increase	2133	0.96 (0.92–1.01)	2041	0.88 (0.84–0.92)	0.0066
AHEI-2010					
Greatest decline	246	1.12 (0.96–1.30)	310	1.33 (1.16–1.52)	
Moderate decline	288	1.07 (0.93–1.23)	261	1.00 (0.87–1.15)	
Stable	842	1.00 (ref.)	814	1.00 (ref.)	
Moderate increase	339	0.98 (0.87–1.12)	294	0.89 (0.78–1.02)	
Greatest increase	418	1.01 (0.89–1.15)	362	0.97 (0.85–1.10)	
Per 1 SD increase	2133	0.96 (0.91–1.01)	2041	0.90 (0.85–0.95)	0.1617
aMED					
Greatest decline	437	1.12 (0.97–1.29)	481	1.29 (1.12–1.48)	
Moderate decline	401	1.07 (0.93–1.22)	365	0.97 (0.84–1.11)	
Stable	441	1.00 (ref.)	450	1.00 (ref.)	
Moderate increase	402	1.07 (0.93–1.23)	357	0.95 (0.82–1.10)	
Greatest increase	452	1.09 (0.94–1.25)	388	0.94 (0.81–1.08)	
Per 1 SD increase	2133	0.99 (0.94–1.04)	2041	0.89 (0.84–0.95)	0.1224
DASH					
Greatest decline	218	1.38 (1.18–1.62)	227	1.30 (1.11–1.51)	
Moderate decline	240	1.18 (1.02–1.37)	252	1.17 (1.01–1.34)	
Stable	964	1.00 (ref.)	963	1.00 (ref.)	
Moderate increase	339	1.09 (0.96–1.24)	316	1.07 (0.94–1.21)	
Greatest increase	372	1.07 (0.94–1.21)	283	0.92 (0.80–1.05)	
Per 1 SD increase	2133	0.94 (0.89–0.99)	2041	0.92 (0.87–0.96)	0.6647

AHEI, Alternative Healthy Eating Index; aMED, alternate Mediterranean Diet score; DASH, Dietary Approaches to Stop Hypertension; HEI, Healthy Eating Index. ^1^ Greatest decline: ≥1 SD decrease; moderate decline: 0.5–1 SD decrease; stable: <0.5 SD change; moderate increase: 0.5–1 SD increase; greatest increase: ≥1 SD increase. ^2^ Adjusted for race and ethnicity, age, BMI, history of hypertension, education, marital status, physical activity, menopausal hormone therapy use for women only, BMI change between the two surveys, total energy intake, baseline diet quality score, and smoking model. For HEI-2015 and DASH, the models were further adjusted for alcohol consumption. All variables were from the 10-year follow-up survey, except for race and ethnicity, education, and baseline diet quality score from the baseline questionnaire. ^3^ Based on the Wald test of cross-product terms of sex and the continuous variable for the dietary index, adjusting for the covariates in the multivariate model.

**Table 3:** HRs (95% CI) for cardiovascular disease mortality per 1 SD increment in diet quality over 10 years by subgroups in the Multiethnic Cohort Study.

		HEI-2015	AHEI-2010	aMED	DASH
Subgroups	CVD Death	HR (95% CI) ^1^	HR (95% CI) ^1^	HR (95% CI) ^1^	HR (95% CI) ^1^
Men					
Race and ethnicity					
African American	274	0.94 (0.82–1.08)	0.99 (0.86–1.15)	1.02 (0.88–1.18)	0.90 (0.77–1.04)
Japanese American	688	0.97 (0.89–1.06)	0.99 (0.90–1.08)	1.02 (0.92–1.12)	0.99 (0.90–1.08)
Latino	419	0.90 (0.80–1.00)	0.97 (0.86–1.08)	0.94 (0.83–1.07)	0.88 (0.79–0.99)
Native Hawaiian	126	0.98 (0.80–1.19)	0.90 (0.73–1.11)	0.82 (0.65–1.03)	0.89 (0.73–1.09)
White	626	1.00 (0.91–1.09)	0.92 (0.84–1.01)	1.01 (0.92–1.12)	0.94 (0.86–1.04)
*P* for heterogeneity ^2^		0.3856	0.5888	0.1247	0.4624
Age at 10-year follow-up					
<65 years	234	1.00 (0.86–1.16)	0.95 (0.81–1.10)	1.00 (0.84–1.18)	0.89 (0.76–1.04)
65–74 years	616	0.91 (0.83–0.99)	0.95 (0.86–1.04)	0.95 (0.86–1.05)	0.89 (0.81–0.98)
≥75 years	1283	0.99 (0.92–1.05)	0.97 (0.91–1.04)	1.00 (0.94–1.08)	0.97 (0.91–1.04)
*P* for heterogeneity ^2^		0.4139	0.2707	0.2501	0.0391
Body mass index at 10-year follow-up					
<25 kg/m^2^	885	0.96 (0.89–1.04)	0.97 (0.89–1.05)	1.02 (0.94–1.12)	0.93 (0.86–1.01)
25–30 kg/m^2^	868	0.97 (0.89–1.05)	0.98 (0.90–1.06)	0.97 (0.89–1.05)	0.95 (0.88–1.03)
≥30 kg/m^2^	380	0.99 (0.88–1.11)	0.92 (0.81–1.04)	1.00 (0.87–1.14)	0.96 (0.85–1.08)
*P* for heterogeneity ^2^		0.7485	0.2386	0.2440	0.7195
Smoking status at 10-year follow-up					
Never	709	0.95 (0.87–1.04)	0.96 (0.88–1.05)	1.01 (0.92–1.11)	0.93 (0.85–1.02)
Ever	1424	0.97 (0.91–1.03)	0.96 (0.90–1.02)	0.98 (0.92–1.05)	0.94 (0.88–1.00)
*P* for heterogeneity ^2^		0.9804	0.6452	0.1742	0.9198
Hypertension					
Without hypertension	719	0.97 (0.89–1.06)	1.06 (0.97–1.15)	1.05 (0.96–1.16)	0.97 (0.89–1.06)
With hypertension	1414	0.96 (0.90–1.02)	0.92 (0.86–0.97)	0.96 (0.90–1.03)	0.92 (0.87–0.98)
*P* for heterogeneity ^2^		0.7577	0.0059	0.2536	0.1871
Women					
Race and ethnicity					
African American	360	0.82 (0.73–0.92)	0.76 (0.67–0.86)	0.79 (0.69–0.91)	0.89 (0.78–1.00)
Japanese American	642	0.90 (0.82–0.98)	0.92 (0.83–1.02)	0.89 (0.80–0.99)	0.99 (0.90–1.08)
Latino	313	0.93 (0.83–1.06)	1.01 (0.88–1.15)	1.00 (0.86–1.15)	0.97 (0.84–1.10)
Native Hawaiian	121	0.77 (0.62–0.94)	0.91 (0.73–1.13)	0.90 (0.70–1.16)	0.93 (0.76–1.14)
White	605	0.92 (0.84–1.01)	0.91 (0.83–1.00)	0.91 (0.82–1.01)	0.84 (0.76–0.93)
*P* for heterogeneity ^2^		0.3776	0.1909	0.7574	0.1906
Age at 10-year follow-up					
<65 years	176	0.82 (0.69–0.96)	0.85 (0.71–1.01)	0.77 (0.63–0.93)	0.75 (0.63–0.90)
65–74 years	483	0.95 (0.85–1.05)	0.91 (0.82–1.01)	0.92 (0.82–1.03)	0.99 (0.89–1.11)
≥75 years	1382	0.87 (0.82–0.92)	0.91 (0.85–0.97)	0.90 (0.84–0.97)	0.92 (0.86–0.98)
*P* for heterogeneity ^2^		0.1161	0.4538	0.2496	0.0742
Body mass index at 10-year follow-up					
<25 kg/m^2^	1108	0.88 (0.82–0.94)	0.92 (0.85–0.99)	0.89 (0.83–0.97)	0.90 (0.84–0.97)
25–30 kg/m^2^	555	0.85 (0.77–0.94)	0.86 (0.77–0.95)	0.85 (0.76–0.95)	0.92 (0.83–1.02)
≥30 kg/m^2^	378	0.94 (0.84–1.05)	0.93 (0.82–1.05)	0.98 (0.86–1.12)	0.97 (0.85–1.09)
*P* for heterogeneity ^2^		0.3601	0.1366	0.2537	0.7224
Smoking status at 10-year follow-up					
Never	1255	0.90 (0.84–0.95)	0.92 (0.86–0.99)	0.92 (0.85–0.99)	0.93 (0.87–0.99)
Ever	786	0.86 (0.79–0.93)	0.87 (0.80–0.94)	0.85 (0.78–0.94)	0.89 (0.82–0.97)
*P* for heterogeneity ^2^		0.6351	0.4875	0.3065	0.8453
Hypertension					
Without hypertension	608	0.92 (0.84–1.02)	0.94 (0.85–1.03)	0.89 (0.80–0.99)	0.94 (0.85–1.04)
With hypertension	1433	0.86 (0.81–0.91)	0.88 (0.83–0.94)	0.90 (0.84–0.96)	0.91 (0.85–0.97)
*P* for heterogeneity ^2^		0.2886	0.4189	0.9976	0.8884

AHEI, Alternative Healthy Eating Index; aMED, alternate Mediterranean Diet score; DASH, Dietary Approaches to Stop Hypertension; HEI, Healthy Eating Index. ^1^ Adjusted for race and ethnicity, age, BMI, history of hypertension, education, marital status, physical activity, menopausal hormone therapy use for women only, BMI change between the two surveys, total energy intake, baseline diet quality score, and smoking model. For HEI-2015 and DASH, the models were further adjusted for alcohol consumption. All variables were from the 10-year follow-up survey, except for race and ethnicity, education, and baseline diet quality score from the baseline questionnaire. ^2^ Tests for heterogeneity by subgroups were based on the Wald statistics for cross-product terms of continuous variables for diet quality change and subgroup indicators.

## Data Availability

The data presented in this study are available upon request from the Multiethnic Cohort (MEC) Study. The data are not publicly available because they contain protected health information. All data requests will be reviewed by the MEC Study Research Committee (see https://www.uhcancercenter.org/for-researchers/mec-data-sharing, accessed on 1 July 2023).

## Supplementary Materials

The following supporting information can be downloaded at: https://www.mdpi.com/article/10.3390/nu15153482/s1, Table S1: Characteristics of participants according to change in Alternative Healthy Eating Index (AHEI)-2010 over 10 years in the Multiethnic Cohort Study; Table S2: Characteristics of participants according to change in alternate Mediterranean Diet (aMED) over 10 years in the Multiethnic Cohort Study; Table S3: Characteristics of participants according to change in Dietary Approaches to Stop Hypertension (DASH) over 10 years in the Multiethnic Cohort Study.
