# Supplementary material for: Changes in Diet Quality over 10 Years and Subsequent Mortality from Cardiovascular Disease in the Multiethnic Cohort Study
Source: Nutrients. 2023 Aug 7;15(15):3482. doi: 10.3390/nu15153482 (PMC10421371; doi:10.3390/nu15153482)
Supplement: Supplementary file 1 [file nutrients-15-03482-s001.zip › nutrients-2513010-supplementary.pdf]

Supplementary Tables

Supplementary Table S1. Characteristics of participants according to change in Alternative Healthy Eating Index (AHEI)-2010 over 10 years in the Multiethnic Cohort Study

	Change in Alternative Healthy Eating Index (AHEI) – 2010[1]				
	Greatest decline	Moderate decline	Stable	Moderate increase	Greatest increase
**Men, n**	2,614	3,085	10,468	4,599	5,858
AHEI-2010 score at baseline	72.1 ± 8.8	69.1 ± 9.1	65.8 ± 9.2	62.6 ± 8.8	58.6 ± 8.9
AHEI-2010 score at 10-year follow-up	57.2 ± 9.0	61.9 ± 9.1	66.0 ± 9.2	69.9 ± 8.8	74.5 ± 8.9
Age at baseline (years)	58.8 ± 8.3	58.2 ± 8.4	57.8 ± 8.3	56.9 ± 8.4	56.1 ± 8.0
Age at 10-year follow-up (years)	69.7 ± 8.2	69.1 ± 8.4	68.7 ± 8.3	67.8 ± 8.3	67.1 ± 7.9
Race and ethnicity (%)					
African American	11.6	10.0	7.9	6.8	6.0
Japanese American	24.2	28.4	35.5	39.2	44.3
Latino	27.3	23.8	19.2	15.4	13.1
Native Hawaiian	5.4	5.8	7.1	8.3	7.8
White	31.5	31.9	30.2	30.3	28.8
Education (%)					
≤High school	34.7	29.6	28.2	26.4	26.4
Vocational school	29.7	30.4	30.2	31.2	31.8
≥Graduated college	35.6	40.1	41.6	42.4	41.9
Body mass index at baseline (kg/m^2)	26.6 ± 3.8	26.4 ± 3.7	26.3 ± 3.8	26.4 ± 3.7	26.4 ± 3.8
Body mass index at 10-year follow-up (kg/m^2)	26.9 ± 4.4	26.8 ± 4.2	26.6 ± 4.2	26.6 ± 4.2	26.5 ± 4.1
Smoking status at baseline (%)					
Never	36.0	36.3	38.2	38.2	34.3
Former	51.3	50.5	49.5	48.6	51.2
Current	12.7	13.3	12.3	13.1	14.6
Smoking status at 10-year follow-up (%)					
Never	36.2	36.4	38.3	38.4	34.3
Former	55.7	55.8	54.6	54.9	58.8
Current	8.1	7.8	7.2	6.7	6.9
Physical activity at baseline (h/d)	1.5 ± 1.6	1.5 ± 1.5	1.5 ± 1.5	1.5 ± 1.5	1.5 ± 1.5
Physical activity at 10-year follow-up (h/d)	1.5 ± 1.6	1.6 ± 1.6	1.6 ± 1.6	1.7 ± 1.7	1.8 ± 1.7
Alcohol intake at baseline (g/day)	12.2 ± 19.7	12.7 ± 22.9	12.9 ± 25.4	15.4 ± 29.2	20.4 ± 36.4
Alcohol intake at 10-year follow-up (g/day)	12.1 ± 24.8	11.5 ± 21.8	10.7 ± 20.3	12.1 ± 20.5	12.5 ± 17.7
History of hypertension at either survey	52.9	53.0	52.4	51.9	52.8
**Women, n**	3,472	4,058	13,522	6,019	7,666
AHEI-2010 score at baseline	72.3 ± 8.4	69.7 ± 8.5	67.0 ± 8.6	64.0 ± 8.3	60.1 ± 8.3
AHEI-2010 score at 10-year follow-up	58.2 ± 8.6	62.9 ± 8.5	67.2 ± 8.7	71.0 ± 8.3	75.1 ± 8.2
Age at baseline (years)	59.0 ± 8.4	58.3 ± 8.3	57.8 ± 8.2	56.7 ± 8.2	55.4 ± 8.0
Age at 10-year follow-up (years)	70.0 ± 8.4	69.2 ± 8.3	68.8 ± 8.2	67.7 ± 8.2	66.5 ± 8.0
Race and ethnicity (%)					
African American	17.5	14.8	11.4	9.7	8.9
Japanese American	22.3	29.2	35.1	39.0	39.0
Latino	23.6	19.5	16.2	13.1	10.7
Native Hawaiian	6.2	6.5	7.7	8.0	8.9
White	30.4	30.0	29.6	30.1	32.4
Education (%)					
≤High school	39.6	37.1	34.4	31.0	27.4
Vocational school	31.9	31.7	31.7	32.4	33.3
≥Graduated college	28.6	31.1	33.9	36.6	39.3
Body mass index at baseline (kg/m^2)	26.2 ± 5.2	25.7 ± 5.0	25.5 ± 5.1	25.3 ± 4.9	25.3 ± 5.0
Body mass index at 10-year follow-up (kg/m^2)	26.7 ± 5.7	26.2 ± 5.5	26.0 ± 5.5	25.7 ± 5.2	25.7 ± 5.3
Smoking status at baseline (%)					
Never	56.1	60.5	63.1	63.2	60.2
Former	31.4	29.5	26.4	27.2	28.1
Current	12.6	10.0	10.6	9.6	11.7
Smoking status at 10-year follow-up (%)					
Never	56.4	60.7	63.2	63.2	60.3
Former	35.9	33.1	31.3	32.0	34.3
Current	7.7	6.2	5.5	4.8	5.4
Physical activity at baseline (h/d)	1.2 ± 1.3	1.2 ± 1.3	1.2 ± 1.3	1.3 ± 1.3	1.2 ± 1.3
Physical activity at 10-year follow-up (h/d)	1.3 ± 1.4	1.3 ± 1.4	1.4 ± 1.4	1.5 ± 1.5	1.5 ± 1.5
Alcohol intake at baseline (g/day)	5.4 ± 12.6	4.3 ± 11.0	4.2 ± 12.4	4.8 ± 15.4	5.8 ± 17.3
Alcohol intake at 10-year follow-up (g/day)	4.9 ± 13.9	3.9 ± 11.1	3.7 ± 10.3	3.8 ± 9.4	4.6 ± 9.7
History of hypertension at either survey	55.4	54.1	52.1	50.2	48.8

[1]Greatest decline: ≥ 1 SD decrease; moderate decline: 0.5-< 1 SD decrease; stable: < 0.5 SD change; moderate increase: 0.5-< 1 SD increase; greatest increase: ≥ 1 SD increase.

Supplementary Table S2. Characteristics of participants according to change in alternate Mediterranean Diet (aMED) over 10 years in the Multiethnic Cohort Study

	Change in alternate Mediterranean Diet (aMED)[1]				
	Greatest decline	Moderate decline	Stable	Moderate increase	Greatest increase
**Men, n**	4,915	4,829	5,963	5,146	5,771
aMED score at baseline	5.6 ± 1.4	4.8 ± 1.6	4.3 ± 1.7	3.9 ± 1.6	3.0 ± 1.4
aMED score at 10-year follow-up	3.0 ± 1.4	3.8 ± 1.6	4.3 ± 1.7	4.9 ± 1.6	5.6 ± 1.4
Age at baseline (years)	58.1 ± 8.4	57.9 ± 8.5	57.4 ± 8.2	57.1 ± 8.3	56.7 ± 8.1
Age at 10-year follow-up (years)	69.1 ± 8.4	68.8 ± 8.5	68.4 ± 8.2	68.1 ± 8.2	67.6 ± 8.0
Race and ethnicity (%)					
African American	9.2	8.7	7.6	7.2	7.3
Japanese American	32.7	36.0	36.0	37.6	38.2
Latino	18.9	16.6	19.0	18.6	19.2
Native Hawaiian	7.3	7.4	7.6	6.6	6.8
White	32.0	31.3	29.8	30.1	28.5
Education (%)					
≤High school	29.5	28.6	28.9	27.9	26.7
Vocational school	30.0	30.5	30.5	31.6	30.8
≥Graduated college	40.4	40.9	40.6	40.5	42.5
Body mass index at baseline (kg/m^2)	26.4 ± 3.8	26.4 ± 3.8	26.4 ± 3.7	26.3 ± 3.8	26.3 ± 3.8
Body mass index at 10-year follow-up (kg/m^2)	26.7 ± 4.3	26.7 ± 4.3	26.7 ± 4.2	26.6 ± 4.2	26.6 ± 4.2
Smoking status at baseline (%)					
Never	36.8	38.2	37.0	36.9	35.8
Former	50.3	49.1	50.2	50.2	50.2
Current	12.9	12.7	12.9	13.0	14.1
Smoking status at 10-year follow-up (%)					
Never	36.8	38.3	37.1	37.0	36.0
Former	55.8	54.5	55.4	56.1	57.1
Current	7.4	7.2	7.5	6.9	6.9
Physical activity at baseline (h/d)	1.5 ± 1.5	1.5 ± 1.5	1.5 ± 1.5	1.5 ± 1.5	1.4 ± 1.5
Physical activity at 10-year follow-up (h/d)	1.5 ± 1.6	1.6 ± 1.7	1.7 ± 1.6	1.7 ± 1.6	1.7 ± 1.7
Alcohol intake at baseline (g/day)	14.7 ± 27.3	14.6 ± 28.5	14.4 ± 27.3	15.2 ± 29.2	15.6 ± 29.3
Alcohol intake at 10-year follow-up (g/day)	10.8 ± 20.4	10.9 ± 20.1	11.3 ± 20.5	11.9 ± 21.1	12.6 ± 20.2
History of hypertension at either survey	53.7	53.9	52.2	52.1	51.1
**Women, n**	6,445	6,191	7,819	6,669	7,613
aMED score at baseline	5.5 ± 1.4	4.7 ± 1.6	4.3 ± 1.7	3.7 ± 1.6	2.9 ± 1.4
aMED score at 10-year follow-up	2.9 ± 1.4	3.7 ± 1.6	4.3 ± 1.7	4.7 ± 1.6	5.5 ± 1.4
Age at baseline (years)	58.4 ± 8.4	57.9 ± 8.3	57.5 ± 8.2	56.8 ± 8.2	56.1 ± 8.1
Age at 10-year follow-up (years)	69.4 ± 8.3	68.8 ± 8.4	68.4 ± 8.2	67.8 ± 8.2	67.1 ± 8.1
Race and ethnicity (%)					
African American	13.7	11.7	10.7	10.9	11.1
Japanese American	32.5	34.7	35.9	36.0	34.0
Latino	15.4	14.5	15.1	15.8	16.9
Native Hawaiian	7.9	7.8	7.5	8.1	7.4
White	30.5	31.3	30.8	29.2	30.6
Education (%)					
≤High school	36.0	33.9	32.7	32.4	31.1
Vocational school	31.1	32.3	32.8	32.4	32.2
≥Graduated college	32.8	33.8	34.6	35.1	36.7
Body mass index at baseline (kg/m^2)	25.8 ± 5.1	25.6 ± 5.1	25.5 ± 5.0	25.4 ± 5.1	25.3 ± 4.9
Body mass index at 10-year follow-up (kg/m^2)	26.2 ± 5.5	26.1 ± 5.5	25.9 ± 5.4	25.9 ± 5.4	25.8 ± 5.2
Smoking status at baseline (%)					
Never	60.0	61.3	61.9	62.1	61.7
Former	29.4	28.4	27.3	26.8	27.2
Current	10.6	10.4	10.7	11.1	11.0
Smoking status at 10-year follow-up (%)					
Never	60.3	61.4	62.1	62.2	61.9
Former	33.9	32.9	32.1	32.1	33.0
Current	5.9	5.8	5.9	5.7	5.1
Physical activity at baseline (h/d)	1.2 ± 1.3	1.2 ± 1.3	1.2 ± 1.3	1.2 ± 1.3	1.2 ± 1.3
Physical activity at 10-year follow-up (h/d)	1.3 ± 1.4	1.4 ± 1.5	1.4 ± 1.4	1.5 ± 1.5	1.5 ± 1.5
Alcohol intake at baseline (g/day)	5.0 ± 13.9	4.8 ± 13.6	4.8 ± 14.0	4.6 ± 14.1	4.7 ± 14.4
Alcohol intake at 10-year follow-up (g/day)	4.1 ± 11.1	4.0 ± 10.8	4.1 ± 10.9	4.0 ± 10.4	4.1 ± 9.6
History of hypertension at either survey	54.6	53.0	51.4	50.8	48.8

[1]Greatest decline: ≥ 1 SD decrease; moderate decline: 0.5-< 1 SD decrease; stable: < 0.5 SD change; moderate increase: 0.5-< 1 SD increase; greatest increase: ≥ 1 SD increase.

Supplementary Table S3. Characteristics of participants according to change in Dietary Approaches to Stop Hypertension (DASH) over 10 years in the Multiethnic Cohort Study

	Change in Dietary Approaches to Stop Hypertension (DASH)[1]				
	Greatest decline	Moderate decline	Stable	Moderate increase	Greatest increase
**Men, n**	1926	2609	12582	4337	5170
DASH score at baseline	28.1 ± 3.6	26.4 ± 3.8	24.6 ± 4.2	22.8 ± 4.2	21.1 ± 3.9
DASH score at 10-year follow-up	21.6 ± 3.6	23.0 ± 3.8	24.7 ± 4.2	26.3 ± 4.2	28.0 ± 3.9
Age at baseline (years)	58.6 ± 8.7	58.2 ± 8.5	57.6 ± 8.3	57.0 ± 8.2	56.4 ± 7.9
Age at 10-year follow-up (years)	69.6 ± 8.7	69.2 ± 8.5	68.5 ± 8.3	68.0 ± 8.2	67.5 ± 7.8
Race and ethnicity (%)					
African American	11.5	9.4	8.1	6.9	6.3
Japanese American	27.3	29.4	33.9	40.3	44.9
Latino	22.4	18.9	18.7	18.5	16.4
Native Hawaiian	6.5	6.7	7.1	7.0	7.8
White	32.3	35.6	32.2	27.2	24.6
Education (%)					
≤High school	31.6	27.6	27.3	27.8	30.2
Vocational school	31.0	30.1	30.1	31.7	31.5
≥Graduated college	37.4	42.3	42.6	40.5	38.3
Body mass index at baseline (kg/m^2)	26.5 ± 3.8	26.4 ± 3.8	26.3 ± 3.8	26.4 ± 3.8	26.5 ± 3.7
Body mass index at 10-year follow-up (kg/m^2)	27.0 ± 4.5	26.8 ± 4.3	26.6 ± 4.2	26.6 ± 4.2	26.5 ± 4.1
Smoking status at baseline (%)					
Never	36.2	35.9	37.6	37.8	35.0
Former	51.8	51.5	49.6	48.5	50.9
Current	12.0	12.6	12.8	13.7	14.1
Smoking status at 10-year follow-up (%)					
Never	36.2	36.0	37.7	38.0	35.1
Former	56.0	55.5	55.1	54.8	58.5
Current	7.8	8.5	7.1	7.2	6.4
Physical activity at baseline (h/d)	1.5 ± 1.5	1.5 ± 1.5	1.5 ± 1.5	1.5 ± 1.5	1.4 ± 1.5
Physical activity at 10-year follow-up (h/d)	1.5 ± 1.7	1.6 ± 1.6	1.7 ± 1.6	1.7 ± 1.6	1.7 ± 1.7
Alcohol intake at baseline (g/day)	12.8 ± 26.2	15.4 ± 29.5	14.6 ± 27.3	14.9 ± 28.1	16.2 ± 30.9
Alcohol intake at 10-year follow-up (g/day)	10.3 ± 21.6	12.4 ± 22.2	11.6 ± 20.6	11.2 ± 18.9	11.7 ± 20.1
History of hypertension at either survey	54.6	53.0	51.8	52.1	53.6
**Women, n**	2666	3440	16274	5759	6598
DASH score at baseline	28.1 ± 3.5	26.5 ± 3.8	24.7 ± 4.1	23.1 ± 4.0	21.4 ± 3.9
DASH score at 10-year follow-up	21.7 ± 3.5	23.1 ± 3.9	24.9 ± 4.1	26.5 ± 4.0	28.3 ± 3.8
Age at baseline (years)	59.2 ± 8.4	58.7 ± 8.4	57.6 ± 8.3	56.7 ± 8.1	55.6 ± 7.9
Age at 10-year follow-up (years)	70.1 ± 8.5	69.6 ± 8.4	68.5 ± 8.3	67.7 ± 8.1	66.6 ± 7.9
Race and ethnicity (%)					
African American	15.9	15.2	11.7	9.5	9.3
Japanese American	26.8	30.4	33.3	38.5	40.1
Latino	18.9	16.5	15.5	14.4	15.2
Native Hawaiian	8.2	7.4	7.3	7.5	8.9
White	30.1	30.5	32.2	30.1	26.5
Education (%)					
≤High school	37.8	36.1	32.5	32.0	32.2
Vocational school	31.7	31.4	32.3	31.2	33.4
≥Graduated college	30.5	32.5	35.2	36.8	34.4
Body mass index at baseline (kg/m^2)	26.2 ± 5.2	25.8 ± 5.1	25.5 ± 5.0	25.2 ± 4.8	25.5 ± 5.1
Body mass index at 10-year follow-up (kg/m^2)	26.7 ± 5.8	26.2 ± 5.5	26.0 ± 5.4	25.7 ± 5.2	25.8 ± 5.3
Smoking status at baseline (%)					
Never	58.3	61.8	61.2	62.4	62.4
Former	31.1	28.0	28.1	27.0	26.2
Current	10.7	10.2	10.7	10.7	11.4
Smoking status at 10-year follow-up (%)					
Never	58.5	62.0	61.3	62.5	62.5
Former	35.0	31.6	32.8	32.0	33.0
Current	6.5	6.4	5.8	5.5	4.6
Physical activity at baseline (h/d)	1.2 ± 1.2	1.2 ± 1.3	1.2 ± 1.3	1.2 ± 1.3	1.2 ± 1.3
Physical activity at 10-year follow-up (h/d)	1.2 ± 1.3	1.4 ± 1.5	1.4 ± 1.4	1.5 ± 1.5	1.5 ± 1.5
Alcohol intake at baseline (g/day)	4.8 ± 14.1	4.6 ± 13.0	5.0 ± 14.5	5.0 ± 14.9	4.2 ± 12.5
Alcohol intake at 10-year follow-up (g/day)	4.1 ± 10.9	4.0 ± 10.3	4.3 ± 11.0	4.0 ± 9.9	3.5 ± 9.8
History of hypertension at either survey	55.7	55.0	52.0	49.4	49.3

[1]Greatest decline: ≥ 1 SD decrease; moderate decline: 0.5-< 1 SD decrease; stable: < 0.5 SD change; moderate increase: 0.5-< 1 SD increase; greatest increase: ≥ 1 SD increase.
